# Phylogenetic analysis and victim contact tracing of rabies virus from humans and dogs in Bali, Indonesia

**DOI:** 10.1017/S0950268813002021

**Published:** 2013-08-19

**Authors:** G. N. K. MAHARDIKA, N. DIBIA, N. S. BUDAYANTI, N. M. SUSILAWATHI, K. SUBRATA, A. E. DARWINATA, F. S. WIGNALL, J. A. RICHT, W. A. VALDIVIA-GRANDA, A. A. R. SUDEWI

**Affiliations:** 1 The Animal Biomedical and Molecular Biology Laboratory, Faculty of Veterinary Medicine Udayana University, Bali, Indonesia; 2 Disease Investigation Center, Directorate General Livestock and Animal Health, Jl Sesetan, Denpasar, Bali, Indonesia; 3 Microbiology Department, Faculty of Medicine Udayana University, Bali, Indonesia; 4 Neurology Department, Faculty of Medicine Udayana University, Bali, Indonesia; 5 Bali Provincial Health Office, Denpasar, Bali, Indonesia; 6 Oxford University Clinical Research Unit, Ho Chi Minh City, Vietnam; 7 Kansas State University, Diagnostic Medicine/Pathobiology, Manhattan, KS, USA; 8 Orion Integrated Biosciences Inc., New Rochelle, NY, USA

**Keywords:** Bali, nucleoprotein, phylogenetic, rabies virus, sequence, timeline

## Abstract

The emergence of human and animal rabies in Bali since November 2008 has attracted local, national and international interest. The potential origin and time of introduction of rabies virus to Bali is described. The nucleoprotein (N) gene of rabies virus from dog brain and human clinical specimens was sequenced using an automated DNA sequencer. Phylogenetic inference with Bayesian Markov Chain Monte Carlo (MCMC) analysis using the Bayesian Evolutionary Analysis by Sampling Trees (BEAST) v. 1.7.5 software confirmed that the outbreak of rabies in Bali was caused by an Indonesian lineage virus following a single introduction. The ancestor of Bali viruses was the descendant of a virus from Kalimantan. Contact tracing showed that the event most likely occurred in early 2008. The introduction of rabies into a large unvaccinated dog population in Bali clearly demonstrates the risk of disease transmission for government agencies and should lead to an increased preparedness and efforts for sustained risk reduction to prevent such events from occurring in future.

## INTRODUCTION

Rabies disease is an almost always fatal infection of the central nervous system which is caused by rabies virus (RABV) and occurs in various animal species as well as in humans [1]. The virus is a member of *Lyssavirus* genus of the Rhabdoviridae family [1, 2]. RABV has an exceptionally strong tropism for the central nervous system, travelling through axon fibres [3]. Rabies is a longstanding worldwide human health problem [4] and is estimated to cause up to 60 000 human deaths annually. However, the number could be much higher as a study in Tanzania reported that the number of deaths could be as much as 100 times higher than the number of officially reported cases [5]. Rabies also causes a worldwide economic burden due to the estimated 10 million people seeking expensive post-exposure prophylaxis (PEP) treatment after being bitten by a suspected rabid animal [2]. Although always fatal in unvaccinated individuals, rabies is almost always preventable if proper PEP management is provided [6].

RABV belongs to the order of viruses containing a single negative-strand RNA genome known as the Mononegavirales [7]. The 12 kb genome acts a template for the generation of anti-genomic positive-strand RNA and mRNAs. The latter are translated into structural proteins N, P, M, G and L, and some additional non-structural proteins [8, 9]. The N protein is the main component of the nucleocapsid. The P protein is a viral polymerase co-factor. The M protein shapes the virion and facilitates budding release from infected cells. The G protein is a glycoprotein that forms a homotrimer with surface spikes and contributes to cell receptor attachment. The last L protein is an RNA-dependent RNA polymerase that is a pivotal enzyme for mRNA generation and genome replication (reviewed in [3]).

Formerly known as a rabies-free area, since November 2008 Bali has been experiencing an outbreak of rabies throughout the province. There have been a number of human fatalities as a result of dog bite incidents [8]. The outbreak has also led to wide concern in the local community and the demand for human and animal anti-rabies vaccine (ARV) and rabies-immunoglobulin (RIG) has exceeded both provincial and national stocks. The outbreak has wider impact outside Bali as travellers to this popular tourist location might need pre-exposure prophylaxis before they travel to Bali or require PEP if exposed or bitten after arriving in Bali [9–11].

Little or no rigorous scientific investigation of rabies outbreaks in formerly rabies-free areas has been peformed. This research may be particularly valuable in preventing such events from occurring in the future. The origin, genetic variation and the introduction of RABV to dogs in Bali requires investigation to better understand how rabies may have been introduced to Bali and the reason for the sudden occurrence of human deaths due to rabies.

Fortunately, a sequence database of RABVs from various animals and islands in Indonesia was available in GenBank [12]. The nucleoprotein gene fragment has been used to map the molecular epidemiology of RABV worldwide [13]. We analysed the nucleoprotein gene fragment of RABV from clinical specimens from dogs and humans to determine the possible source and time of rabies introduction.

## METHODS

### Samples and ethical clearance

The source of clinical samples for this study were dog brains that were confirmed as rabies positive using the FAT test. Human samples were the post-mortem CSF specimens of rabies victims that were confirmed as positive by RT–PCR [8]. The district of origin and collection year of the isolates were Badung (H01RK/2009, H06KR/2010, D137/2009, D147/2010); Tabanan (H02BL/2009, H05KW/2010); Buleleng (H04WA/2010, D144/2010); and Karangasem (H03MS/2009, D140/2010). Four recent isolates from other islands, namely Sumatra (SM066-09/2010), Sulawesi (SL417-09/2009), Flores (FL007-09/2009) and Java (JA001-08/2008), were included. Ethical clearance for the use of human samples was obtained from the Research Ethics Committee of the Faculty of Medicine, Udayana University, Denpasar, Bali, Indonesia, reference number 723/Skrt/XI/2010 dated 12 November 2010 [10], and was consistent with the principles of the Declaration of Helsinki. The patients' families agreed that the samples could be collected and that patients' data could be included in this research and provided written informed consent. Dog brain samples were obtained from the collection of The Animal Biomedical and Molecular Biology Laboratory Faculty of Veterinary Medicine, Udayana University. Approval for the use of animal samples was granted by The Ethics Commission for the Use of Animals in Research and Education of the Faculty of Veterinary Medicine, Udayana University, Bali Indonesia, reference number 04/KE-PH/IV/2010, in accordance with chapter 7.8. of the Use of Animals in Research and Education of Terrestrial Animal Health Code of the World Organization for Animal Health.

### Primer sets

Three primer sets were selected to generate overlapping cDNA of the RABV nucleoprotein fragment gene using the sequence database of RABVs isolated in Indonesia available in GenBank. The primer3 program, available online (http://www.embnet.sk/cgi-bin/primer3_www.cgi) was used. Following alignment of the selected primers with the database using MEGA5 [14], degenerative nucleotides were assigned for respective residues found to be polymorphic. The primers used in this study are listed in Supplementary Table S1 (available online).

### RT–PCR and sequencing

Genomic RNA isolation, RT–PCR and sequencing were performed as previously described [8]. Sequences were aligned using MEGA5 software [14]. Each final sequence was based on the consensus sequence of at least two independent RT–PCR products. The secondary sequence data mined from GenBank and used as reference sequences are listed in Table 1. Different phylogenetic analysis and Bayesian phylogenetic analyses were deployed to define specific lineages of RABV for comparative analyses of evolutionary rates. The maximum clade credibility (MCC) phylogeny was inferred in the Bayesian Markov Chain Monte Carlo (MCMC) analysis using the Bayesian Evolutionary Analysis by Sampling Trees (BEAST) software package version 1.7 (http://beast.bio.ed.ac.uk) after Drummond *et al.* [15]. An extensible mark-up language (XML) file of the data was generated from the sequence nexus format using Bayesian Evolutionary Analysis Utility (BEAUti) software. Definitive year of isolation of each sequence was entered as tips date if information was available in GenBank. The deployed substitution model was the Hasegawa, Kishino & Yano (HKY) model. The trace quality was tested using Tracer v. 1.5 software (http://tree.bio.ed.ac.uk/software/tracer/). Sample trees generated in BEAST were transformed into a single tree using TreeAnnotator. The final tree was generated using FigTree v. 1.4.0 software (http://beast.bio.ed.ac.uk/FigTree). To test hypothetical ancestors of Bali viruses, the most recent common ancestor (MRCA) was analysed with pre-defined taxon subsets of Bali viruses with members of the respective clade.

**Table 1.** List of secondary sequence data used in this study for phylogeographical analysis

Country and origin area	ID number	Year of isolation	Accession number	Ref.
Indonesia, Sumatra	03003INDO	2003	EU086192	[30]
	SN0014	2000	AB154235	[12]
	SC02-90	2002	AB154243	
	SS01-21	2001	AB154238	
	SC01-70	2001	AB154242	
	SN00-03	2000	AB154234	
	SC97-01	1997	AB154233	
	SS01-13	2001	AB154237	
	SC00-12	2000	AB154225	
	SC00-45	2000	AB154227	
	SC00-36	2000	AB154226	
	SC01-63	2001	AB154228	
	SC02-89	2002	AB154231	
	SC02-83	2002	AB154230	
	SC02-91	2002	AB154232	
	SC02-79	2002	AB154229	
	SC01-66	2001	AB154213	
	SC01-65	2001	AB154214	
	SC02-87	2002	AB154224	
	SC01-74	2001	AB154209	
	SC01-68	2001	AB154208	
	SC01-75	2001	AB154210	
	SC02-82	2002	AB154211	
	SC01-73	2001	AB154212	
	SN00-14	2000	AB154235	
	SC02-90	2002	AB154243	
	SS01-21	2001	AB154238	
Indonesia, Kalimantan	KL97-03	1997	AB154223	[12]
	KL00-18	2000	AB154221	
Indonesia, Sulawesi	SW97-04	1997	AB154241	[12]
	SW02-22	2002	AB154240	
	SW01-11	2001	AB154239	
Indonesia, Flores	FL01-06	2001	AB154215	[12]
	FL01-08	2001	AB154216	
	FL01-27	2003	AB154222	
	Fl02-10	2002	AB154217	
	FL97-01	1997	AB154218	
Indonesia, West Java	JA97-05	1997	AB154220	[12]
China	Guizhou_Qx5	2003	DQ666296	[31]
Thailand	8738THA	n.a.	U22653	[32]
Sri Lanka	1294	1986	AY238549	[33]
Canada	91RABN2756	n.a.	L20674	[34]
Russia	9141RUS	1988	U22656	[32]
Mexico	9126MEX	1991	U22477	[32]
Namibia	9227NAM	1992	U22649	[32]
Poland	8618POL	n.a.	U22840	[32]
Philippines	Phi152-14	n.a.	AB070772	[35]

n.a., Not available.

### Contact tracing

To investigate the arrival of RABV in Bali, a survey was conducted which included personal interviews with community members and healthcare workers who were involved with the index human case [8]. A questionnaire was developed to trace bite events and possible rabies-related human deaths. The questionnaire included potential rabies-related deaths following dog bites and clinical signs of rabies in the biting dogs. The recorded clinical signs were hypersalivation, hydrophobia, aerophobia, photophobia, pain or paraesthesia at the bite site, agitation, convulsion and paralysis. The interview was done with all village and sub-village leaders as well as with medical professionals (doctors and nurses) in the Kuta-Selatan sub-district community health centre and two supporting community health centres. There are six villages in the Kuta Selatan sub-district: Jimbaran, Benoa, Tanjung Benoa, Kutuh, Ungasan and Pecatu, and 68 sub-villages. Suspect cases were further confirmed by interviews with the patient's family.

## RESULTS

A fragment of the nucleoprotein gene of RABV obtained from five dogs and six human clinical samples was sequenced. The length of readable cDNA sequence of the animal viruses varied from 892 to 1148 bp. The cDNA sequences obtained in this study have been submitted to GenBank (accession nos. JQ768453-58, JX462633, JX462634, JX462639, JX462643, KF482528-32).

**Fig. 1.**
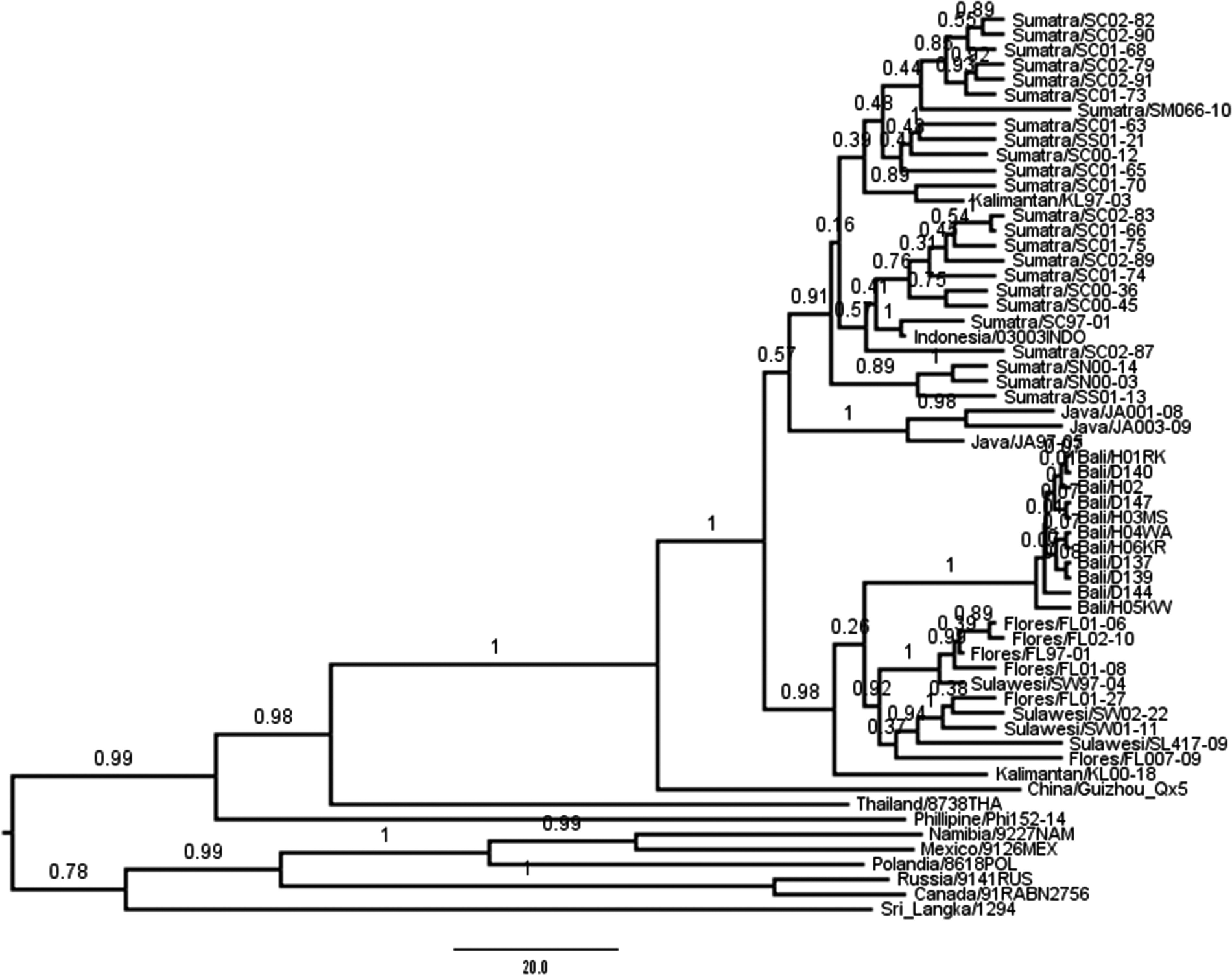
The maximum clade credibility phylogeny for nucleoprotein gene fragment of rabies virus from the outbreak in Bali in 2009–2010 analysed with sequence data of some viruses from other islands in Indonesia and from some countries that are available in GenBank. The name of the island and the isolate code is presented for rabies viruses from Indonesia. The letters ‘H’ and ‘D’ for viruses from Bali represent human and dog viruses, respectively. Phylogeny was inferred in the Bayesian MCMC analysis using BEAST software. The posterior probability values are shown next to respective node.

All viruses detected in animals and humans in Bali were closely related to each other with a genetic distance of 0·0004. The MCC tree of phylogenetic analysis of the 892 bp fragments of RABV nucleoprotein genes from animals and humans during the recent outbreak in Bali and street viruses from various countries is presented in Figure 1. The results show that the ancestor of Bali viruses was the descendant of Kalimantan 00-18 strain. The viruses form a common clade with those from Sulawesi and Flores that is significantly separated from Java, Sumatra, and another Kalimantan strain (Kalimantan KL 97-03) with 100% posterior probability value. The trace analysis to test time to MRCA (TMRCA) of the various hypothetical origins of Bali viruses of Kalimantan 00-18, Sulawesi or Flores, shows that Kalimantan 00-18 is stongly related to Bali viruses and estimated the mean generation time as 28·8 years [s.e. = 0·3415, 95% highest posterior density (HPD) interval = 18·2–40·3, effective sample size = 286·8141]. The tree also show that Kalimantan KL 00-18 was the ancestor for Sulawesi and Flores viruses.

A list of events linked to the introduction of rabies in Bali is presented in Table 2. Interviews with community members and healthcare workers involved with the index human rabies case revealed that there were at least three other deaths with clinical signs of rabies associated with a history of dog bite in the immediate vicinity and around the same time. The index case (patient KW) was bitten by a rabid dog on 16 September 2008 and died on 23 November 2008. Besides this case, other possible cases were L (female, 4 years), MA (male, 32 years), and MORP (male, 3 years) who died on 13, 14 and 21 November 2008, respectively, and who were bitten by rabid dogs on 6 September, 9 September, and 19 October 2008, respectively. After the index case, patient TA (male, 32 years) was admitted to hospital on 15 January and died on 16 January 2009. He was bitten during July 2008. The bite sites of all cases were anterior and posterior extremities. Assuming that these were the first human cases and that they were probably related to the introduction of a rabid dog, by calculating 90 days as the longest incubation period for most cases of canine rabies, we estimated that the introduction of RABV into Bali was most likely in early 2008. The community was not aware of any suspected rabies cases as the number of bite cases increased.

**Table 2.** Cases of unreported deaths with clinical signs of rabies and history of dog bite as well as the bite incidences in the sub-district Kuta Selatan, Badung district, Bali, Indonesia from June 2008 to 30 November 2008 when the rabies outbreak was formally declared

January–April 2008	Estimated arrival of rabies virus in Bali
July 2008	Victim TA bitten in Ungasan
August 2008	Dog bite incident in Ungasan; patient healthy
6 September 2008	Victim L (female, 4 years) bitten in Ungasan
9 September 2008	Victim MA (male, 32 years) bitten
16 September 2008	Index case KW (male, 28 years) bitten in Ungasan
17 September 2008	Victim L died
19 October 2008	Patient MORP (male, 3 years) bitten in Ungasan
14 November 2008	Victim MA died
21 November 2008	Victim MORP died
23 November 2008	Index case KW died
26 November 2008	Bite incident in Kedonganan. The dog died and was positive by FAT
30 November 2008	Rabies outbreak formally declared in Bali
16 January 2009	Victim TA died

## DISCUSSION

An understanding of how RABVs enter a rabies-free area may prove invaluable in preventing the future spread of RABV from one island or one country to another. It is especially pertinent for archipelagos such as Indonesia. Molecular epidemiology provides a more accurate and relatively simple tool to answer this question compared to serology or other conventional methods [16, 17]. Although closely related, the Indonesian RABVs have a nucleotide homology ranging from 94% to 100% [12]. The time of virus isolation and identification is also crucial to the molecular epidemiological investigations. Indonesian RABV data available in GenBank date from 1997 to 2003.

Phylogeography of RABVs from around the world shows distinct differences. RABVs from Asia, Africa and Europe have been divided into subgroups of Arctic, Europe, Africa, Middle East, South Asia, Malay and South East Asia [13]. Indonesian viruses form a common cluster with viruses from China and the Philippines [12].

These results also show that the outbreak was most likely caused by a single incursion. The samples analysed in this study represent human and dog viruses from four out of nine Bali districts collected during the peak of the outbreak between 2009 and 2010 [8]. The analysis shows that all animal and human RABVs obtained from Bali were homogeneous. The genetic distance between all analysed viruses was negligible.

Conducting rabies forensic tests to predict the origin of the causative outbreak is paramount in preventing such an event in the future. The advance of statistical inference brings a new insight for a virus forensic study. As the virus, especially RNA viruses such as influenza and rabies [18], undergoes a high mutation rate, its geographical footprint and former dispersal are imprinted in its genome [19, 20]. This approach allows researchers to reconstruct a quantitative spatio-temporal dispersal of any virus [18]. The recommended software to determine such event is BEAST [21]. Using this analysis, Lemey *et al.* [22] were able to reconstruct the influenza and RABV dispersal patterns while accommodating phylogenetic uncertainty.

Using the protocol, it was discovered that the ancestor of Bali viruses was the descendant of Kalimantan 00-18 strain, but not significantly separated from those of Sulawesi and Flores. Multiple MCMC analysis have also been conducted to test TMRCA with a pre-defined taxon subset if the Bali viruses were closely related to Kalimantan 00-18, Sulawesi or Flores viruses. The valid estimate was found in Kalimantan 00-18 and the Bali subset. This result supports the tree analysis that the Bali outbreak was caused by a Kalimantan descendant, which is correspondingly the ancestor of Sulawesi and Flores viruses.

However, the actual generation time, and therefore, geographical origin of the Bali outbreak remains a mystery. The lower bound of the HPD Kalimantan–Bali subset was around 18 years as shown by TMRCA analysis. This means that the Bali virus ancestor diverged a decade before the actual isolation year of the putative ancestor. The clade support for the divergence of Kalimantan to Bali and Sulawesi-Flores strains is indeed very strong (posterior probability of 98%, Fig. 1); however, the partition of its descendants into Bali and Sulawesi-Flores has no significant support. In other word, the Bali viruses are not separable from those of Sulawesi and Flores. The protocol is likely to require current sequences from all over Indonesia. The huge archipelagic nature of the country and the limitation of transport infrastructure in large islands such as Kalimantan, Sulawesi, and Flores restrict the natural circulation of the virus, so the drifting event of its genome will be independent in one island or even in one district to another in one single island. Moreover, the almost 10-year span of the collection of isolates throughout Indonesia has, due to lack of surveillance in every district in an endemic island except Sumatra, curbed the power of any genetic software, including the BEAST platform, to delineate the real geographical origin of the virus. The common detection probabilities of RABV surveillance in endemic counties were calculated as *P* < 0·1 [23], which decreases the chance of locating the actual source of the epidemic.

Kalimantan and Sulawesi are indeed the most plausible hypothetical origin of the Bali strains. Kalimantan and Sulawesi are known to be rabies-endemic islands for decades [24], while Flores has been infected by RABV since 1990s following infected dog transportation from Sulawesi. The circulating virus in Flores was predicted to have originated from Sulawesi [25] and was confirmed by genetic analysis [12]. This event is claimed to have been repeated, in that that the Bali virus was from Sulawesi [24].

From an anthropological point of view, Kalimantan and Sulawesi appear to be a reasonable origin of Bali's RABV. There is a strong belief in the country that some tribes in those islands, especially the Bugis tribe, are real sea people [26]. They usually build settlements close to or, even, on the water. Originating from Sulawesi, the tribe is spread throughout Indonesia, especially Kalimantan, and sustains its culture by sea voyaging and living close to water. That the location of index case lies at the southern end of Bali is another clue of RABV introduction by fishermen from Kalimantan or Sulawesi. Ungasan village, where the index human and animal cases occurred [8], is close to a major fish market of Bali at Kedonganan village. The village is beyond animal quarantine control. The Bugis tribe is known to have settled at the surrounding villages a long time ago. The fishermen who sell their catch harvest at the market are from Java, Madura, and Sulawesi, as well as local Bali. Bali's major national and international harbour is also located in the Peninsula. However, it is unlikely to be the entry point of the RABV since it is controlled by the Bali Animal Quarantine Office.

If the Bali RABV was, indeed, introduced from Kalimantan or Sulawesi, this would be a repeat of events where rabies was introduced to Flores from Sulawesi in 1997. The mode of introduction was thought to be via fishermen who brought an infected dog to the East Flores Island [25].

It is likely that the virus was transferred to Bali via a similar route. Legal and illegal transportation of dog and cats has been found to be a major risk of rabies introduction in island regions like Taiwan [27]. However, the possible motives for moving an infected dog to Bali need to be investigated. Dogs may serve as sailing companions, may be used for trade or food. Some people believe that fishermen use dogs as alarms for unfavourable weather conditions while sailing and that this may be the most likely reason for them to be brought on board. This is unproven and there is no evidence or surveys which show that fishermen regularly transport dogs between islands. The event appears to be very irregular. In community interviews, very few people were aware that there might have been inter-island dog transport. For fish transport, large ships usually anchor offshore of Kedonganan village, and the fish is transported to the beach using small vessels. The presence of a dog will not be observed unless the ships are inspected. Dogs on fishing boat transport has been estimated as the carrier for inter-island transmission to Terengganu-Malaysia, Flores, and even Bali [23, 25, 28].

The transport of dogs from other islands to Bali for business purposes cannot be ignored. Transport of dogs, cats and monkeys to Bali has been banned for a long time to keep the province rabies free. However, the regulation has not been properly enforced and public participation has been lacking. Illegal transport occurs mostly at Gilimanuk Harbour, which is the entry point for ferries from Java. Given that the circulating virus in Bali was not closely related to viruses from Java, it is unlikely that rabies was introduced through dog trafficking for commercial purposes.

Another possibility is the use of dogs for food. Balinese traditionally do not eat dog meat but there are some tribes in Indonesia that do consume dog meat. Some tribes, including tribes from Sulawesi, serve dog meat at parties as a special treat for guests and some people from dog meat-eating tribes have settled in Bali and introduced the dog-eating practice to the locals. Many small restaurants now offer dog meat in Bali, especially in Badung, the site of the index human case, Denpasar and Singaraja. Bali is the likely source of dogs for most restaurants. It is possible that some fishermen might have transported an infected dog to Bali as a gift for relatives for dog meat consumption at a party. Importation of dogs for the meat trade has been claimed as the source for the introduction of rabies in Maluku [23].

Although it is not a listed bioterrorism agent [29], rabies has indeed caused panic in Bali due to the 100% case-fatality rate [8]. Rabies could be used as a bioterrorism agent simply to create panic. Bali is a well-known terrorist target in Indonesia with two previous terrorist bombing events. The only clue in such circumstances would be if the virus originated from a known terrorist area. This study did not find any reason to suggest that bioterrorism was a potential motive for the introduction of rabies to Bali.

RABV was most likely to have been introduced to Bali in early 2008. We found that there have been other deaths with clinical signs of rabies and a history of dog bite at the same time and area as the index case. The earliest dog bite incident causing clinical signs and death from rabies was recorded early in September 2008. However, after the index case, a 32-year-old male who was bitten around July 2008 and died on 16 January 2009 was also confirmed as positive [8]. Assuming that the most cases of canine rabies have an incubation period of 14–90 days [2], especially if the bite site is in the extremities as happened in all possible human rabies cases found this study, the introduction of RABV to Bali is likely to have happened in early 2008. Given that dog density is very high in Bali [8], with most animals roaming free in the neighbourhood, dog bite is not an uncommon event. The community did not suspect rabies as the number of bite cases increased. The information found via contact tracing regarding possible human rabies deaths before the index case occurred is believed to be highly accurate. The main respondents were all village and sub-village leaders from the index case's sub-district. The social system in Bali is unique, especially when dealing with the death of a village or sub-village member. All sub-village members are well informed of the circumstances surrounding the death and burial or cremation will be performed by the sub-village community, not just by the family.

The likelihood that this represents a repeat of events where rabies-infected dogs have been transported from one island to another, as seen from Sulawesi to Flores [12, 25], and now probably to Bali, is a strong signal for government agencies to increase their preparedness so that such events will not recur in the future. Rabies alerts must be in place in rabies-free islands close to endemic areas. As a multi-island nation, every beach is accessible to fishermen from other islands with a small boat or dugout vessel. Where the risk is high, a barrier vaccination programme might need to be considered. The lessons learnt from the incursion into Bali are extremely valuable and as a new outbreak, it is likely that there were many deaths due to rabies before it was diagnosed. The victims would have been unaware of the rabies risk and wound management and the medical system would have been ill-prepared with inadequate stocks of vaccine and RIG [8]. Public and healthcare provider awareness as well as rabies vaccine and RIG stocks are crucial in preventing human deaths from rabies in disease-free areas.

Risk reduction efforts should equally take place in endemic islands and districts not only in new outbreak areas as widely practised in Indonesia. Regarding the fact that dogs are the main carriers of rabies in the most parts of the world [1, 2], as shown in Bali [8], as well as the proof in this study that human and dog RABVs are genetically homogenous, and the nature of the disease is of a highly variable incubation period [2], the risk of transporting a rabies-incubating dog from an endemic area to a free area remains high.

Having a long stretch of beaches, quarantine control of animal transport at every beach is hardly possible in Indonesia. Adequate knowledge, awareness and behaviour regarding rabies risks should be made available in the free, at risk, as well as endemic areas in Indonesia. When it comes to inter-island transport, all dogs should have been vaccinated properly.

In conclusion, the outbreak of rabies in Bali was caused by a virus from Indonesia following a single introduction, and most likely occurred in early 2008. The lessons learned from this introduction of rabies into a large unvaccinated dog population provide a clear demonstration of the risks for government agencies and call for an increased preparedness to prevent such events from occurring in the future. Movement of people and their pets throughout the archipelago mean that virus introduction may occur in other areas. The occurrence of rabies in Bali has shown that public and healthcare provider awareness, dog vaccination programmes and good stocks of human anti-rabies and immunoglobulin are all crucial in preventing human rabies deaths.

## Supplementary Material

Supplementary Material Supplementary information supplied by authors.

## Supplementary material

For supplementary material accompanying this paper visit https://doi.org/10.1017/S0950268813002021. click here to view supplementary material
