# Supplementary material for: Phylogenetic analysis and victim contact tracing of rabies virus from humans and dogs in Bali, Indonesia
Source: Epidemiol Infect. 2013 Aug 19;142(6):1146–54. doi: 10.1017/S0950268813002021 (PMC4045169; doi:10.1017/S0950268813002021)
Supplement: Supplementary Material — Supplementary information supplied by authors. [file S0950268813002021sup001.docx]

Supplementary Table S1. *List of oligonucleotide primers used in this study*

| Oligonucleotide name | Sequence (5’-3’) |
| --- | --- |
| NF36Y | TCAGGTGGTCTCYTTGAAGCC |
| NR587 | TTGGCACACATCTTGTGAGT |
| NF303R | CCGATGTRGAAGGGAGTTGG |
| NR836 | ACGAACGGAAGTGGATGAAA |
| NF587 | ACTCACAAGATGTGTGCCAA |
| NR1251 | CTTTAGTCGACCTCCGTTCA |
